# Survey on the presence of antibiotic residues in raw milk samples from six sites of the dairy pool of Niamey, Niger

**DOI:** 10.14202/vetworld.2019.1970-1974

**Published:** 2019-12-14

**Authors:** Amadou Morou Madougou, Caroline Douny, Nassim Moula, Marie-Louise Scippo, Véronique Delcenserie, Georges Daube, Marichatou Hamani, Nicolas Korsak

**Affiliations:** 1 Department of Food Science, Fundamental and Applied Research for Animal and Health, Faculty of Veterinary Medicine, University of Liège, Sart-Tilman, B43b Liège, B-4000 Belgium; 2 Laboratoire Central de l’Elevage (LABOCEL), Niamey, Niger; 3 Department of Animal Production Faculté d’Agronomie, Université Abdou Moumouni de Niamey, Niger

**Keywords:** antibiotic residues, cow, Delvotest, Niger, raw milk

## Abstract

**Background and Aim:**

Antibiotics are widely used in animal production for treating the diseases and for preventing or increasing animal growth. The presence of antibiotic residues in milk is a public health problem. The aim of this study was to assess the use of antibiotic residues in raw milk from the dairy pool of Niamey in three farms (Toukounous, Kirkissoye, and Niamey) and three collection centers (Hamdallaye, Kollo, and Say).

**Materials and Methods:**

A direct interview (questionnaire) was used to collect data regarding the mode of use of antibiotics, the level of knowledge of farmers according to the withdrawal period, and a cross-sectional study was conducted on 192 samples of raw milk. The Delvotest^®^ T was used to monitor antibiotic residues in milk. The data were analyzed using SAS and R software.

**Results:**

The most commonly used antibiotics were those from the family of tetracycline (86.7%) and from the family of beta-lactams (13.3%). Regarding the statements of farmers, the reasons why the farmers use antibiotics were the following: About 47% in case of prevention and treatment, 29% for treatment, 12% for prevention, and 12% for increase dairy production. Moreover, the farmers lacked the necessary information about withdrawal period. Screening of antibiotic residues was performed using a standardized biological test kit, the Delvotest^®^. In total, from 192 samples of raw milk, 19 (9.9%) were positive including ten from collection centers and nine from farms. This could lead to a risk of exposure when a consumer drinks locally produced raw milk.

**Conclusion:**

Raw milk supplied from the area of the study has a level of antibiotic residues, and the breeders have a low level of knowledge about the withdrawal period.

## Introduction

Milk occupies an increasingly important place in the daily diet of people in the world in general and sub-Sahara Africa in particular. Raw milk is an essential material of many dairy products (yogurt and Degué). In Niger, milk is a strategic component for nutritional and economic reasons [1]. The production results essentially from traditional breeding cows belonging to breeds such as Azawak, Goudali, Bororo, Djelli, and Kouri. In these farms, health treatment is performed empirically, involving indiscriminate use and uncontrolled veterinary medicines including antibiotics [2].

Residues in food from animal origin can constitute a risk for the consumer by initiating the following side effects: Allergic outcomes (penicillin and streptomycin), poisoning (chloramphenicol), modification of the intestinal flora (tetracycline), and selection of antibiotic-resistant bacteria [2,3].

The uncontrolled use of antimicrobial drugs in general and antibiotics in particular in animal production can lead to the occurrence of residues in the dairy products processed from these animals, especially when the withdrawal period is not respected [2,4-6]. Besides, antibiotic residues in animal products will be the factor of environmental and water pollution. The administration of antibiotics imposes a withdrawal period, which is the time required for the animal body to remove completely chemical residue from treatment outcome. This period is specified on the instructions for the use of veterinary drugs, as long as, the product is authorized in the country. During this time, any use or consumption of meat, eggs, and milk is prohibited [7]. During the withdrawal period, animal slaughtering for human consumption is prohibited as well as delivering, for human consumption, products issued from these animals such as eggs and milk [7].

According to the harmful effects of veterinary drugs for humans, no residue monitoring program exists at present despite the fact that the demand for raw milk is constantly increasing in Niger. Veterinary drugs are also available, easily accessible to farmers, and their distribution is not controlled by the government authorities. Unfortunately, the data of antibiotics residue in milk are still nonexisting in Niger, and screening the residues in milk is urgently needed.

The aim of this study was to estimate the percentage of residual antimicrobial drugs in raw milk of the cow in different areas of Niger. Specifically, it was initially highlights the percentage of knowledge of the importance of use of antibiotics in the sites of Toukounous, Hamdallaye, Niamey, Kollo, Kirkissoye, and Say by survey then, to assess the prevalence of residual antimicrobial drugs in raw milk of the cow using a standardized biological test kit form, the Delvotest^®^ T (DSM Food Specialities, Delft, Netherlands).

## Materials and Methods

### Ethical approval

Ethical approval is not needed for such type of study.

### Informed consents

Informed consent was obtained from each participant.

### Study area and period

The study was carried out in six sites, which are three farms (Toukous, Kirkissoye, and Niamey) and three collection centers (Hamdallaye, Kollo, and Say) from the dairy pool of Niamey (Niger) during the rainy season from July 2015 to September 2015. The survey questionnaires were administered to a total of 15 breeders: Twelve at collection centers (four collectors by center) and three in the farms (one in each farm). The choice of 15 breeders was based on the presence of the collector who was present during the period of survey and the manager of the farms. Milk collection is performed under the supervision of organizations of breeders around a collection center. Each organization is represented by a collector who collects milk with a motorcycle in 3-4 villages around a radius of 40 km to the center of Hamdallaye, 35 km for Kollo, and 15 for Say. The collector is the interface between the milk collection center and the breeders. The survey was performed at the rainy season during which few breeders continued to supply the collection centers because of the agricultural fieldwork.

The farm managers at the three farms were selected because they manage the organization, health, and sale of milk for the benefit of other farm members from Kirkissoye, Niamey, and Toukounous. The information has been collected on the types of antibiotics used, diseases encountered, general purpose of the antibiotics use, and the knowledge of the breeders about withdrawal period.

### Sampling of raw milk

The study is a cross-sectional one and consisted of making samples from bulk tank milk in the three collection centers and three farms. Random cluster sampling method was used to increase the representativeness of the study; we assumed a prior prevalence of 25% contamination of milk with antibiotics residues based on prior studies. This value was based on a similar study conducted in Ivory Coast where Kouamé [8] observed a prevalence of 24.7% of antibiotics residues in milk. The desired precision was fixed at 5% for a confidence interval of 95%.

The sample size based on prior studies [8] was calculated by the Thrusfield formula [9], which is:

N = p* (1-p)*Z^2^/i^2^

Where: N is the sample size; Z is the Z value (e.g., 1.96 for 95% confidence level, confidence interval 5%); p is the percentage picking a choice, expressed as decimal; i is the confidence interval, expressed as decimal.

A total of 192 samples had been collected (the samples were distributed equitably among the six sites to avoid bias during statistical analyses 32 samples for each site) randomly in sterile containers and the milk samples were transferred to the laboratory at Central Laboratory of Livestock (LABOCEL).

### Antimicrobial residues screening test

The cross-sectional study has involved screening of milk for antimicrobial residues using the standard microbiological methods called Delvotest^®^ T (DSM Food Specialities, Delft, Netherlands) under the article number 5500786 and 8618000, respectively, kit 25 tests ZE/EF 25 and Delvotest apparatus 108-A marketed by N.V. International Medical Product S.A (Invoice of July 8, 2015). Ten mL of bulk milk were collected randomly in sterile containers on which the name of the site and number were listed. Delvotest^®^ is based on the inhibition of microbial growth in the presence of antibiotic residues. Hundred mL of milk sample was transferred to the kit containing nutrient agar embedded with *Bacillus stearotherms* spores and bromocresol purple indicator using a micropipette and incubated at 64°C, and the results were recorded for 2-3 h. The residues of microbial growth were identified in the absence of antibiotics; the microorganism uses nutrients, ferments lactose, and produces lactic acid, which results in a change in the color of the bromocresol compound from purple to yellow. If the antibiotic residues exist, the microorganism cannot grow and the purple color of the medium remains unchanged.

### Statistical analysis

The statistical analyses of the data were performed using the software SAS system 2001 (SAS Institute, Inc., Cary, NC) and the R software (version 3.2.5) (Ross Ihaker and Robert Gentlemen, New Zealand) were used for statistical analysis. The significant level was considered at p<0.05.

## Results

### The screening test results

A total of 192 milk samples were collected and tested for both the farms (96 samples) and collection centers (96 samples). Overall, 9.9% (19/192) of milk samples tested were contaminated with antimicrobial drugs (Table-1). There were no significant differences between the farms and collection centers because of p>0.05.

**Table-1:** Antimicrobial residue screening results.

Sites	Positive samples/total (%)	Positive/total by site (%)
Centers		
Say	4/32 (12.5)	10/96 (10.4%)
Kollo	1/32 (3.1)	
Hamdalaye	5/32 (15.6)	
Farms		
Kirkissoye	4/32 (12.5)	9/96 (9.4%)
Niamey	5/32 (15.6)	
Toukounous	0/32 (0)	
Total	19/192 (9.9)	

### Survey about the use of antibiotics

Table-2 gives us information on the choice of antibiotics, the purpose of uses, use of milk, and knowledge of the breeders about withdrawal period.

**Table-2:** Results of the survey as stated by breeders (n=15).

Parameters	Numbers of occurrences=n	%=n/N×100
Types of antibiotics used		
Oxytetracycline	13	86.7
Beta-lactam	2	13.3
Diseases encountered		
Infectious and non-infectious diseases	12	84.2
Metabolic diseases	1	5.2
Others without diagnosis	2	10.5
General purpose of use		
Preventive and curative	7	47
Curative	4	29
Preventive	2	12
Increase dairy production	2	12
Knowledge of the breeders about withdrawal period		
Same day of treatment	5	33.3
Three days after treatment	1	6.7
According to the notice of the drug	5	33.3
One week	2	13.3
No ideas about the withdrawal period	2	13.3
Milk use		
Family consumption and sales	11	76.6
Sales	2	11.7
Milk processing	2	11.7

The families of antibiotics used are mainly tetracyclines (oxytetracycline) (86.7%) followed by beta-lactam antibiotics (penicillin) (13.3%). They are used individually or in combination with vitamins. The diseases which need the use of antibiotics are infectious and non-infectious diseases (84.2%), metabolic diseases (5.2%), and others (10.5%).

According to the survey, the breeders did not respect the withdrawal period which indicates that the consumers of locally produced milk are exposed to the contamination of raw milk with unacceptable concentrations of antimicrobial residues. The highest risk of ingesting residues above the acceptable daily intake levels prescribed by the Codex Alimentarius Commission was deemed to be for beta-lactam or penicillin antibiotics due to their low acceptable daily intake and the high frequency of usage of these antibiotics locally especially in the treatment of infection and promote growth. The milk was used for family consumption and milk sales by 76.6% of breeders and 11.7% breeders used milk, respectively, for sales and processing according to the survey.

## Discussion

For the first time in Niger that a qualitative test is used for a widespread assessment of drug residues present in raw milk collected from three farms and three collection centers in the dairy pool of Niamey. According to international standards, the presence of antibiotics in both milk and milk product is not acceptable because consumers may already have allergic reactions and some health problems [2]. Therefore, the presence of antibiotic residues in raw milk should be controlled because it can upset the process of dairy production and can permit the growth of some pathogens such as *Salmonella* and *Staphylococcus aureus* in milk [2].

The population interviewed during the study had no device to evaluate the quality of the produced milk. They performed only a traditional physical control of the milk (tasting, visual aspect, smell, color, and filtration). The surveyed breeders said that they do not have the resources necessary for quality control. However, the collection centers of Say, Kollo, and Hamdalaye had the device Delvotest^®^ T available for the control of antimicrobial residues at reception.

This study has confirmed that tetracycline was the main group of antibiotics detected in milk samples. Thirteen of the interviewed persons were using antibiotics such as tetracycline and two used the beta-lactams for the treatment of diseases, and this statement is confirmed by other authors [10].

In Côte d’Ivoire, Kouamé [8] showed that tetracycline was generally used to treat sick cows. Furthermore, surveys by Alambedji *et al*. [2] revealed that one of the most used antibiotics in Senegal was also tetracycline, and the same was reported in Nigeria [11].

According to the answers of breeders, the antibiotics were used for preventive, curative, and to increase dairy production. In Belgium, Reybroeck [10] confirms the statements that the antibiotics are used for curative and preventive in a study on the search for antibiotic residues in milk.

Tetracycline imported from foreign countries seems to be the most used antibiotic family in the West African sub-region. However, in Niger, imported veterinary drugs are dominated by antimicrobial compounds (86% of therapeutic classes) among which, 54% are trypanocides. The majority of veterinary products originate from Europe [12]. All surveyed breeders (100%) stated that they used antibiotics in their farms in case of infection [2,7].

According to the state of the breeders, the most antibiotics found on the market are penicillin-based drugs and oxytetracycline on the market [2,8]. Seven out of 15 breeders ignored the importance of the withdrawal period. The majority of interviewed persons did not respect the withdrawal period owing probably to their low education [7,13].

The produced milk was at the same time intended for family consumption, for business, and for processing into dairy products (like the processing of cheese called “Tchoukou”). These results are similar to those of Franck *et al*. [1] who showed that the proportion of milk sold was 68% against 32% intended for auto-consumption in Niger.

Among 192 raw milk samples collected for antimicrobial residue research using the Delvotest T, 19 were positive (9.9%) with no significant difference between farms and collection centers, which indicates that there is not enough control over the use of the antibiotics in Niger. The positive samples at collection centers and farms may be the results of the treatment of animals without respecting the withdrawal period. Another reason may be the addition of antibiotics as preservative by collectors when they have a long distance between the village and collection center [14,15]. The antimicrobial drugs are obtained without any control of authorities but in Belgium (Europe), for example, sales of veterinary products are done with an authorization to market granted by the European Commission, after opinion of the Committee for Medicinal Products for Veterinary Use established within the European Medicines Agency (centralized procedure) [16].

The obtained results of the number of positive milk samples in our study are lower than those observed in Côte d’Ivoire with 24.7% positive samples [8], in Ghana with 35.5% of positive sample [14], in Mali with 16% of positive samples [17], and Benin with 83% positive samples [7] but practically similar to those observed in Mauritania with 11% positive samples [18] using the Delvotest^®^ T.

These results showed that milk contains residues of antibiotics in Niger as well as everywhere in West Africa. The presence of antibiotic residues could be a risk for the consumer so that there is an urgent need for a greater level of awareness among breeders regarding the withdrawal period following antimicrobial therapy. However, the breeding development faces several difficulties, including animal diseases that require the use of veterinary medicines, which can lead to the presence of residues in food of animal origin.

Delvotest^®^ T is a very useful screening test, but it has the disadvantage of neither identifying the molecule nor quantifying it and not confirms the level of antibiotics present in suspicious samples against the maximum residue limits fixed by the regulation [19].

On the other hand, increasing the level of health management of animals by local veterinary doctors and developing – in-house closed rearing systems can improve milk yield and meat production amounts then the control of veterinary drug administration rate. The public health entity for consumer protection which necessitates integration of local veterinary medical organization and human health authorities to support the cost of reducing contamination and discarding contaminated milk and meat by the veterinary medical drugs, as a recent recommendation of the WHO [20].

This study has exposed a potentially serious public health statement for consumers of raw milk in Niger and also repeated exposure over long periods to antimicrobial drug residues in milk can lead to drug resistance within population [14].

We, therefore, recommend further surveillance of milk for the detection of the presence of antibiotic residue and enforcement of the actual legislation on the use of antibiotics to ensure the safety of animal products in Niger.

## Conclusion

In Niger, the breeding sector is a major source in terms of food security of the population and as well as a source of supply of animal protein. However, the breeding development faces several obstacles, including animal diseases that require the use of antimicrobial drugs, which can lead to the presence of residues in food of animal origin. The main factor considered to underlie the presence of antibiotic residues may be unrelated to the variation in the withdrawal period and the level of education. It is in this context that we have undertaken an investigation on the presence of residues in milk. The surveys make global identities of the antibiotics used by farmers and also to appreciate the different systems in connection with the use of antibiotics. Therefore, raw milk at the six sites in the dairy pool of Niamey contains antibiotic residues with a percentage of 9.9%. These results demonstrate the noncompliance of the withdrawal period, abusive use of antimicrobial drugs without any control of authorities, and the study has also revealed the fact that breeders use tetracyclines in 86.7% and 13.3% beta-lactams. There is no sufficient and necessary control over the farms and milk collection centers in determining the remaining antibiotics during the delivery of milk. The poor livestock management, low supervision over the livestock and husbandries, noncompliance with sanitary conditions including the obligatory vaccination can cause infections to the animals and the farmers have no choice other than using antimicrobial drugs for treatment.

On the other hands, Training of the farmers about the withdrawal period after treatment of diseased animals would be a very effective response in reducing antimicrobial residues in raw milk. Moreover, control and detection of antibiotic residues in food and other products of animal origin can also be done to protect the health of consumers. It is recommended that the antimicrobial residue of any kind of antibiotics in Niger must be implemented with standards of the European Union in the absence of internal standards.

Research must be done to clearly define the risk factor of antimicrobial drug residues in raw milk in all the dairy pool of Niger and how it can introduce the respect of withdrawal period following drug administration.

## Authors’ Contribution

AMM: Prepared the questionnaire for data collection, sampling method, prepared the manuscript and made laboratory analysis. CD: Revised the manuscript. NM: Made and approved statistical analysis. MS: Revised the manuscript. VD: Prepared and revised the manuscript. GD: Revised the manuscript. MH: Revised the manuscript. NK: Prepared and revised the manuscript. All authors read and approved the final manuscript.
